# Bacterial Colonization of Orthodontic Devices (Molar Bands, Nance Buttons, and Acrylic Plates) and Its Impact on the Marginal Periodontium and Palatal Fibromucosa in Teenagers: A Cross-Sectional Clinical–Microbiological Study

**DOI:** 10.3390/medicina61091717

**Published:** 2025-09-21

**Authors:** Bianca Dragos, Dana-Cristina Bratu, George Popa, Magda-Mihaela Luca, Remus-Christian Bratu, Cosmin Sinescu

**Affiliations:** 1 Doctoral School, Faculty of Dental Medicine, “Victor Babes” University of Medicine and Pharmacy of Timisoara, 300041 Timisoara, Romania; bianca.roman@umft.ro; 2 Research Centre in Dental Medicine Using Conventional and Alternative Technologies, Faculty of Dental Medicine, “Victor Babes” University of Medicine and Pharmacy of Timisoara, 300070 Timisoara, Romania; 3 Department of Orthodontics II, Orthodontic Research Centre, Faculty of Dental Medicine, “Victor Babes” University of Medicine and Pharmacy of Timisoara, 300041 Timisoara, Romania; 4 Department of Pediatric Dentistry, Pediatric Dentistry Research Center, Faculty of Dental Medicine, “Victor Babes” University of Medicine and Pharmacy of Timisoara, 300041 Timisoara, Romania; luca.magda@umft.ro; 5 Faculty of Dental Medicine, “Victor Babes” University of Medicine and Pharmacy of Timisoara, 300041 Timisoara, Romania; remus.bratu@student.umft.ro; 6 Department of Prostheses Technology and Dental Materials, Research Center in Dental Medicine Using Conventional and Alternative Technologies, Faculty of Dental Medicine, “Victor Babes” University of Medicine and Pharmacy of Timisoara, 300070 Timisoara, Romania; sinescu.cosmin@umft.ro

**Keywords:** adolescent, biofilms, dental plaque, gingivitis, mouth mucosa, orthodontic appliances, palate

## Abstract

*Background*: Orthodontic auxiliaries can create plaque-retentive niches that inflame adjacent soft tissues. We compared bacterial colonization on molar bands, Nance buttons, and acrylic plates and assessed associated periodontal and palatal tissue responses in adolescents. *Methods*: In a cross-sectional study (n = 128; 10–17 years), clinical indices (Plaque Index, Gingival Index, bleeding on probing, probing depth) were recorded at device-influenced teeth. Palatal fibromucosa under palate-contacting devices was graded 0–3 (0 = none, 1 = mild/diffuse, 2 = moderate/confluent, 3 = marked with papillary hyperemia). Swabs from device surfaces, adjacent enamel, and palatal mucosa were cultured for total aerobic counts (log_10_ CFU/cm^2^); Streptococcus mutans burden was quantified by qPCR (log_10_ copies/mL). Group differences and adjusted associations were analyzed. *Results*: Palate-contacting devices harbored greater palatal biofilm and presented higher soft-tissue inflammation than bands. In adjusted models, device type (Nance, acrylic) remained associated with higher Gingival Index independent of measured behaviors and wear duration. Palatal colonization tracked closely with palatal erythema, supporting a local dose–response at the palatal interface. *Conclusions*: Appliance design is associated with distinct colonization patterns and soft-tissue responses; palate-covering acrylic components warrant device-specific hygiene and routine palatal inspection. Selecting designs with better cleansability and reinforcing plate-specific cleaning may mitigate palatal inflammation during treatment.

## 1. Introduction

Orthodontic auxiliaries alter the oral ecological balance by introducing new, often rough and sheltered, colonization substrates that complicate oral self-cleansing and may amplify plaque accumulation and soft-tissue inflammation. Contemporary reviews consistently report that orthodontic treatment, especially when appliances are plaque-retentive, elevates plaque and gingival indices and increases the risk of biofilm-mediated complications, motivating closer surveillance of periodontal endpoints during active therapy [1,2].

High-resolution microbial studies now show that dysbiosis can emerge within weeks of appliance placement, with measurable shifts in community composition, diversity, and functional potential across saliva, supragingival, and subgingival niches. A 2024 synthesis mapped these trajectories and linked longer treatment duration with more pronounced white-spot lesions and gingivitis; notably, malocclusion is common globally (≈56%), so exposure to appliance-related microbial perturbation is widespread [3]. Earlier controlled work also documented increases in salivary mutans streptococci/lactobacilli and acidogenicity within six months of fixed appliance placement, reinforcing the temporal proximity between hardware introduction and ecological change [4].

Device geometry and materials modulate these effects. Stainless-steel bands and acrylic pads/buttons differ in surface energy and microroughness, while palatal coverage (Nance button, removable acrylic plate) reduces salivary shear and aeration, fostering low-flow, nutrient-retentive microhabitats. Comparative clinical evidence indicates that appliances with greater coverage and under-surface stagnation impose a higher biofilm burden and worse gingival outcomes than designs with easier access for hygiene; longitudinal comparisons further suggest that hardware choice conditions both the magnitude and spatial distribution of plaque around index teeth and palatal vault [5,6].

Parallel clinical–microbiological data support a mechanistic link between these microhabitats and tissue response: pilot and cohort studies show that insertion of fixed appliances elevates plaque/gingival indices and bleeding on probing alongside detectable changes in supragingival and subgingival communities; conversely, appliance removal is associated with short-term improvements in gingival health and a partial reversion of subgingival profiles [7,8,9,10]. Beyond bulk biomass, pre-treatment microbial profiling and acidogenic (Stephan curve) kinetics can predict white-spot lesion risk during treatment, underscoring the value of baseline risk stratification when selecting appliance type and hygiene protocols [9,10].

Palate-contacting devices merit special attention. A 2024 narrative review on Nance and transpalatal arch (TPA) anchorage concluded that these appliances do not provide absolute anchorage and frequently elicit palatal tissue discomfort and inflammation, recommending meticulous hygiene and periodic tissue checks [11]. Case-based evidence documents severe palatal reactions—including mucosal necrosis under the acrylic button—presenting with alarming signs such as apparent haematemesis that resolve after appliance removal and antiseptic management, highlighting the clinical relevance of early recognition and load reduction on the palatal fibromucosa [12,13,14,15].

There is limited data regarding sampled device surfaces in adolescents, adjacent enamel, and palatal mucosa while aligning these measures with standardized clinical indices across commonly used appliances (bands, Nance, acrylic plates). Direct comparison of these designs, particularly regarding palatal tissue response—remains limited. Therefore, the study aim was to compare device-, enamel-, and palatal-site colonization and associated periodontal/palatal outcomes among molar bands, Nance buttons, and acrylic plates in adolescents. Our null hypothesis is as follows: there are no device-related differences in (i) colonization across sampled niches or (ii) clinical indices; and after adjustment, device biofilm is not associated with gingival inflammation.

## 2. Materials and Methods

### 2.1. Study Design, Setting, Participants, and Calibration

The study rationale was to characterize design-linked colonization niches and their clinical correlates at a single time-point in adolescents, informing prevention strategies and powering future longitudinal work. The cross-sectional design precludes causal inference.

This was a single-visit, cross-sectional study conducted at the “Victor Babeș” University of Medicine and Pharmacy from Timișoara. Throughout the investigation, all procedures adhered to the Declaration of Helsinki and the EU General Data Protection Regulation; the institutional review board approved secondary analysis of de-identified data. Study was approved by the institutional review board (protocol code 038, 28 June 2025). Records were anonymized at extraction, secured on an encrypted server and accessed only by the study team. Written informed consent was obtained from all participants. Participant confidentiality was ensured through anonymized identifier codes and a database.

Consecutive patients aged 10–17 years in active treatment were screened. Eligibility required ≥1 month of continuous wear with a dominant appliance element—molar bands (n = 44), Nance button (n = 41), or removable acrylic plate (n = 43)—and absence of (i) systemic conditions affecting periodontal health, (ii) antibiotics or professional prophylaxis within 30 days, and (iii) ongoing periodontal therapy.

A priori power considered the Gingival Index as the primary endpoint (one-way ANOVA, 3 groups, α = 0.05, f = 0.30 as a moderate effect consistent with reported appliance-related periodontal differences); n ≥ 111 yields 80% power. We enrolled 128 patients. Hygiene/diet were collected via a brief interviewer-administered five-item questionnaire (toothbrushing/day; interdental cleaning; sugary drinks/week; nighttime plate wear; recent mouthrinse use). In a 2-week test–retest subset (n = 20), intraclass correlation coefficients were 0.84 (toothbrushing/day) and 0.81 (sugary drinks/week).

Adolescents were targeted to minimize age-related periodontal variability, reflect the demographic most frequently exposed to these appliances, and reduce confounding from cumulative periodontal breakdown present in adults.

### 2.2. Clinical Examination and Indices

Exams were performed in the morning to prevent diurnal variation. Participants were asked not to alter hygiene on the day of sampling and to avoid eating or brushing for ≥2 h pre-visit. Plaque Index (Silness–Löe; 0–3) and Gingival Index (Löe–Silness; 0–3) were recorded at six sites per index tooth chosen to reflect maximal device influence (e.g., banded first molars; premolars underneath the Nance pad) [16]. Bleeding on probing (BOP%) was computed as bleeding at any of the six sites/total sites × 100, using gentle sulcular probing at the marginal gingiva as recommended for gingivitis assessment [17]. Probing depth (PD, mm) was measured to the nearest 0.5 mm.

Palatal fibromucosa was inspected at three standardized points beneath the acrylic (for Nance and removable plates) or the contralateral palatal vault adjacent to banded molars. Erythema was graded 0–3 (0 = none; 1 = mild, diffuse; 2 = moderate, confluent; 3 = marked with papillary hyperemia). When removable plates were present, patients removed them immediately before inspection to capture the in situ tissue state. Two calibrated examiners independently scored erythema in 20 pilot images and 10 live cases; weighted κ = 0.82 (95% CI 0.69–0.94). For PI and GI, ICC(2,1) = 0.91 and 0.89, respectively.

### 2.3. Microbiological Analysis

Sampling order was device surface → adjacent enamel → palatal mucosa to minimize cross-contamination. Sterile rayon swabs pre-wetted with phosphate-buffered saline (PBS) sampled a 1 cm^2^ region defined by a disposable template. Swabs were rotated with light pressure for 10 s, placed in 1.0 mL PBS, vortexed (30 s), and transported for processing within 2 h.

For bulk biomass, ten-fold serial dilutions were plated in duplicate on tryptic soy agar with 5% sheep blood and incubated at 37 °C, 5% CO_2_ for 48 h; counts were converted to log_10_ CFU/cm^2^. The limit of quantification (LOQ) was 10^2^ CFU per plate; plates below LOQ were imputed as half-LOQ before log-transform. For Streptococcus mutans, Real-time PCR was used [18]. Results were reported as log_10_ copies/mL of suspension. qPCR design, controls, and reporting followed MIQE recommendations (e.g., primer sequences, amplicon length, efficiency, and NTC behavior documented) [19].

Materials/equipment: sterile rayon swabs (COPAN Diagnostics, Murrieta, CA, USA); PBS (Gibco, Thermo Fisher Scientific, Waltham, MA, USA); tryptic soy agar with 5% sheep blood (bioMérieux, Marcy-l’Étoile, France); incubator (Heratherm IMC18, Thermo Fisher, Thermo Fisher Scientific, Waltham, MA, USA); qPCR platform (Applied Biosystems 7500 Fast, Thermo Fisher); SYBR master mix (PowerUp SYBR Green, Thermo Fisher). Orthodontic components: stainless-steel bands (3M Unitek, Monrovia, CA, USA); acrylic resin (Orthocryl, Dentaurum, Ispringen, Germany).

### 2.4. Statistical Analysis

Continuous variables were inspected via histograms/Q–Q plots; homoscedasticity was screened with Levene’s test. Microbial counts and qPCR outputs were analyzed on the log_10_ scale. Group comparisons used one-way ANOVA with Welch correction when variances were unequal. Kruskal–Wallis with Dunn’s post hoc (Holm-adjusted) served as a sensitivity check (reported when discordant). For effect sizes, (ANOVA) and r (nonparametric) were computed with 95% CIs.

Associations were assessed with Spearman’s ρ (exact *p* where n < 50, asymptotic otherwise). A multivariable ordinary least-squares model with HC3 robust standard errors estimated adjusted associations with GI (dependent variable). Predictors were prespecified: device type (bands reference), device biofilm (log_10_ CFU), brushing/day, sugary drinks/week, age, sex, and wear duration (months). Multicollinearity was screened via VIF (<3 acceptable). α = 0.05 (two-sided) defined statistical significance; for multiple pairwise tests, adjusted *p*-values from the Games–Howell/Dunn procedures are reported. Analyses were performed in R 4.3 and Python 3.11.

The protocol conformed to the Declaration of Helsinki and received institutional ethics approval. Participants/guardians provided written informed consent/assent. Reporting follows STROBE guidance for cross-sectional studies, with a completed checklist archived in the study files [20].

Sensitivity covariates included orthodontic treatment stage (leveling/aligning, space-closure/anchorage, finishing), parental education (≤secondary vs. >secondary), and baseline DMFT.

## 3. Results

The three groups were broadly comparable in age (band 15.3 ± 1.9 years; Nance 14.6 ± 1.6 years; acrylic 14.9 ± 2.1 y; *p* = 0.261) and sex distribution (female: 45.6% vs. 63.4% vs. 46.6%; *p* = 0.182), with similar daily toothbrushing frequency (2.1 ± 0.4 vs. 2.1 ± 0.4 vs. 2.2 ± 0.4 times/day; *p* = 0.371) and sugary-drink intake (4.1 ± 1.8 vs. 4.4 ± 1.9 vs. 3.9 ± 2.1 times/week; *p* = 0.410). Wear duration differed significantly (*p* < 0.001), longest in the Nance group (9.6 ± 3.8 months), intermediate in bands (7.4 ± 2.9 months), and shortest in removable acrylic plates (5.6 ± 2.6 months), a spread of 4.0 months between extremes, which we considered in subsequent analyses (Table 1). Key findings: palate-contacting devices exhibited higher palatal colonization and erythema than bands; device type remained associated with gingival inflammation in adjusted models; palatal colonization showed a strong dose–response with palatal erythema.

Biofilm burden differed across devices at every site (all ANOVA *p* < 0.001). Mean device-surface biomass increased from bands (4.6 ± 0.6 log_10_ CFU/cm^2^) to Nance (5.3 ± 0.6) to acrylic plates (5.6 ± 0.6). Adjacent enamel followed the same pattern (3.7 ± 0.6 vs. 4.3 ± 0.4 vs. 4.6 ± 0.6). Palatal mucosa showed the largest separation, with much lower values for bands (3.2 ± 0.4) than Nance (4.8 ± 0.6) and acrylic (4.9 ± 0.7), a difference in ≈1.6–1.7 log_10_ units. *S. mutans* copy number mirrored these gradients (5.2 ± 0.6 vs. 5.9 ± 0.7 vs. 6.2 ± 0.7 log_10_ copies/mL), underscoring greater cariogenic potential in palatal-covering acrylic appliances (Table 2).

Clinical indices varied significantly by device: Plaque Index was highest with Nance (2.1 ± 0.2) versus acrylic (1.7 ± 0.3) and bands (1.6 ± 0.3; *p* < 0.001), and Gingival Index was likewise elevated for Nance (1.7 ± 0.3) relative to bands (1.2 ± 0.3) and comparable to acrylic (1.6 ± 0.3; *p* < 0.001). Bleeding on probing rose from 22.3 ± 11.3% (bands) to 32.9 ± 13.9% (Nance) and 28.9 ± 12.2% (acrylic; *p* = 0.001). Probing depth showed a smaller but significant increase (2.6 ± 0.4 mm bands vs. 2.9 ± 0.4 mm Nance and 2.8 ± 0.6 mm acrylic; *p* = 0.015). Palatal erythema was minimal with bands (0.6 ± 0.4) but higher with Nance (1.6 ± 0.8) and greatest with acrylic plates (1.9 ± 0.7; *p* < 0.001), aligning with the palatal microbial gradients (Table 3).

**Figure 1:**
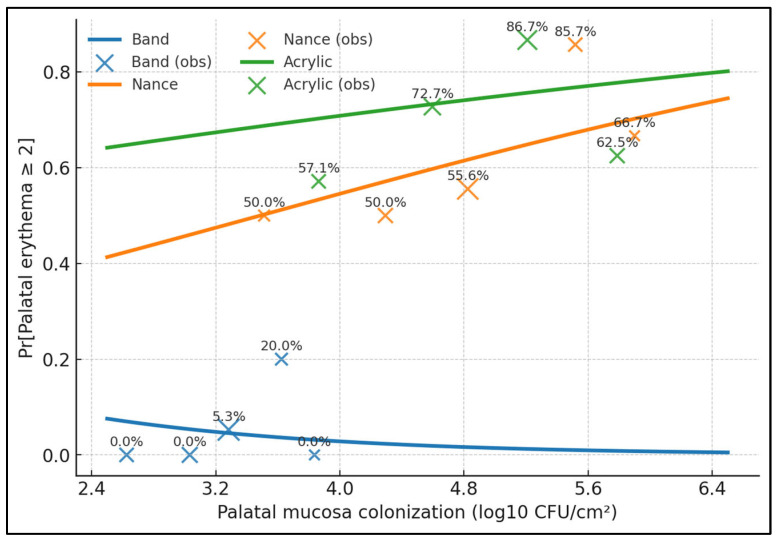
Dose–response of moderate–severe palatal erythema (≥2) versus palatal mucosal colonization (log_10_ CFU/cm^2^), stratified by device. Curves represent predicted probabilities from a logistic model with palatal CFU as the predictor and device-specific smooths; bands (blue) show 95% CIs. Vertical tick marks indicate observed data density. The acrylic and Nance curves rise steeply with increasing palatal colonization, whereas bands remain low across the range.

The acrylic curve sits highest across the entire range, with predicted risk ≈0.66 at 3.8 log10 CFU/cm^2^ rising to ≈0.80 at 6.2; Nance rises from ≈0.42 to ≈0.76 over the same interval. Bands remain near-zero risk, falling from ≈0.07 at 2.5 to ≈0.01 at 6.4, consistent with minimal palatal contact. At a representative colonization of 5.0 log10 CFU/cm^2^, the model estimates Pr(erythema ≥ 2) = 0.73 for acrylic, 0.66 for Nance, and 0.02 for bands—an absolute acrylic–band difference in +0.71 (Figure 1).

When pooling bands and Nance as “fixed” and comparing to removable acrylic plates, removable appliances exhibited higher palatal mucosal colonization (4.9 ± 0.7 vs. 4.1 ± 0.9 log_10_ CFU/cm^2^; *p* < 0.001) and more intense palatal erythema (1.9 ± 0.7 vs. 1.1 ± 0.8; *p* < 0.001). In contrast, overall Gingival Index was similar between categories (both 1.6 on average; *p* = 0.082). These findings indicate that removables disproportionately affect the palatal fibromucosa, while marginal gingival inflammation around teeth is not materially different after pooling by appliance category (Table 4).

Greater device-surface biomass correlated with higher Gingival Index (ρ = 0.3, *p* < 0.001) and slightly deeper probing depths (ρ = 0.2, *p* = 0.029), but not with bleeding on probing (ρ = 0.1, *p* = 0.359). Device biofilm also related to palatal erythema (ρ = 0.4, *p* < 0.001). A strong association was observed between palatal mucosal colonization and erythema (ρ = 0.6, *p* < 0.001), reinforcing a local dose–response at the palatal interface. *S. mutans* load correlated with Gingival Index (ρ = 0.3, *p* = 0.003). Behavioral covariates showed no clear relationships with device biofilm (brush/day ρ = 0.1, *p* = 0.838; sugary drinks/week ρ = 0.1, *p* = 0.106), and wear duration did not track biomass in this cross-section (ρ = 0.1, *p* = 0.993), as seen in Table 5.

**Figure 2:**
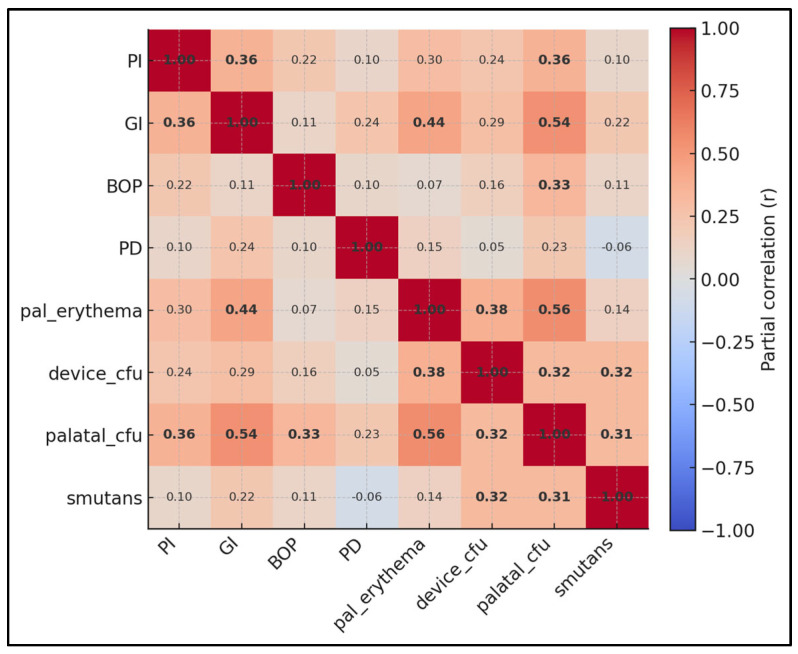
Partial correlation map among clinical and microbiological variables after adjustment for age, sex, toothbrushing, sugary drinks, wear duration, parental education, treatment stage, and baseline DMFT. Warmer hues denote positive correlations. Notably, palatal CFU correlates strongly with palatal erythema, while device biofilm shows modest ties to Gingival Index.

Strongest adjusted associations (bold numbers) include palatal CFU with palatal erythema (r = 0.56), *S. mutans* with GI (r = 0.31), device biofilm with GI (r = 0.29), and palatal CFU with GI (r = 0.54). Gingival and plaque indices remain moderately coupled (PI–GI r = 0.36), and BOP relates to palatal CFU (r = 0.33) more than to device biofilm (r = 0.16), suggesting the mucosal reservoir may contribute to sulcular bleeding beyond device-surface biomass alone. Probing depth shows only weak partial ties (max r = 0.24 with GI), fitting an adolescent cohort where edema outweighs structural change (Figure 2). Nevertheless, device-surface biomass showed modest correlations with Gingival Index and probing depth, whereas palatal colonization displayed a stronger relationship with palatal erythema.

After adjustment for device biofilm, hygiene (brushing/day), sugary drinks, age, sex, and wear duration, appliance type remained the dominant predictor of GI (model R^2^ = 0.3, HC3-robust OLS). Compared with bands, Nance increased GI by 0.6 units (95% CI 0.3 to 0.6; *p* < 0.001) and acrylic by 0.4 units (0.2 to 0.6; *p* < 0.001). In contrast, device-surface biomass (β = 0.1; −0.1 to 0.1; *p* = 0.862), brushing frequency (β = 0.1; *p* = 0.964), sugary drinks (β = 0.1; *p* = 0.667), age (β = 0.1; *p* = 0.450), sex (β = 0.1; *p* = 0.628), and wear duration (β = 0.1; *p* = 0.243) were not significant (Table 6). Findings were robust to adjustment for wear duration, treatment stage, parental education, and baseline DMFT. Device–outcome associations did not differ across wear-duration tertiles (interaction *p*-value = 0.42).

## 4. Discussion

### 4.1. Literature Findings

This study identified device-linked differences in colonization and clinical response in adolescents: palate-contacting devices (Nance, acrylic plates) were associated with greater palatal biofilm and higher palatal erythema than bands, and—in adjusted models—device type remained associated with gingival inflammation. These findings reject the null hypothesis of no between-device differences across sampled niches and clinical indices, while acknowledging that cross-sectional data preclude causal inference. Contextually, evidence from appliance-category comparisons suggests that designs with better cleansability yield more favorable periodontal indices; however, clear aligners were not studied here and this comparison is provided for context only.

Palatal CFU correlated strongly with palatal erythema (ρ = 0.6), outstripping correlations between device CFU and marginal periodontal indices. This highlights the need to examine and record palatal tissues routinely when palatal acrylic is used, not just marginal gingiva. Practical measures include instructing patients to brush the intaglio surface with a dedicated soft brush and allowing periodic “plate-off” intervals during low-risk daytime periods to ventilate tissues, provided biomechanical goals are preserved.

Conventional hygiene metrics (brushing frequency, sugary-drink exposure) had weak or null relationships with colonization in this adolescent cohort. This does not diminish their importance but suggests diminishing returns without device-specific cleaning (interdental brushes around bands; under-surface acrylic cleaning). Scheduling professional prophylaxis to coincide with archwire changes and integrating targeted plaque-disclosing sessions may deliver more impact than generic advice alone for palatal-coverage devices.

Culture and molecular surveys on removable appliances in children have identified complex polymicrobial communities tightly adherent to acrylic, with clinical relevance for mucosal irritation and caries risk [21]. Recent evidence syntheses emphasize that acrylic plates require active disinfection (e.g., effervescent peroxides, chlorhexidine, sodium hypochlorite at safe concentrations) in addition to toothbrushing to meaningfully reduce microbial load; purely mechanical cleaning is often insufficient [22,23]. A controlled in vitro study specific to acrylic-based orthodontic plates further showed that regimented chemical cleaning protocols cut adherent biofilm mass by large margins versus water alone, reinforcing our clinical recommendation for device-specific hygiene coaching when palatal acrylic is used [24].

The fact that appliance type remained independently associated with gingival inflammation even after adjustment for device-surface biomass suggests that design-mediated cleansability and soft-tissue contact mechanics matter as much as (or more than) “how much” plaque is present at a single time-point. Contemporary meta-analyses comparing appliance categories consistently show better periodontal indices with designs that minimize plaque stagnation and allow easier access (e.g., clear thermoplastic aligners) relative to multibracket systems—implicating geometry and access rather than patient behaviors alone [24,25]. Early prospective microbiologic work also demonstrates that fixed appliances can shift salivary bacterial profiles within weeks, preceding clinically meaningful increases in inflammatory indices, which supports the notion that the hardware choice conditions the ecological trajectory of plaque and gingival response [26].

Our gradients in *S. mutans* (highest with palatal acrylic, intermediate with Nance, lowest with bands) mirror prospective data linking appliance design to cariogenic burden. In a 6-month PLOS One study, multibracket appliances yielded markedly higher odds of “risky” salivary *S. mutans* and Lactobacilli counts than clear aligners (odds ratio 7.4 for *S. mutans* at 10^5^ CFU/mL) [27]. A separate prospective cohort likewise found consistently worse plaque and gingival scores with fixed appliances; aligner wear did not increase *S. mutans* over one month, underscoring the role of retentive features and stagnation zones [28]. Beyond appliance category, quantitative PCR studies show early increases in periodontal pathogens during the first weeks after bonding [26] and proportional rises in cariogenic taxa during fixed treatment [29,30,31], which is congruent with our finding that *S. mutans* tracked with the Gingival Index even after accounting for behaviors.

Clinically, the strong dose–response between palatal mucosal colonization and erythema we report (ρ ≈ 0.6) aligns with case-based evidence that palatal acrylic can precipitate severe soft-tissue reactions, including necrosis beneath a Nance button, which resolved after appliance removal and antisepsis [32]. While most literature on inflammatory papillary hyperplasia comes from denture populations, systematic reviews detail how prolonged acrylic contact, reduced ventilation, and micro-trauma predispose to inflamed, hyperplastic palatal tissue—mechanisms that plausibly translate to orthodontic plates with continuous palatal coverage [33]. Mechanistically, co-colonization by *Candida* spp. and *S. mutans* on acrylic can create a highly acidogenic, resilient biofilm matrix that amplifies mucosal inflammation and demineralization risk—again supporting targeted surveillance of the palatal fibromucosa when acrylic components are used [34].

Design features that modulate surface roughness and fluid dynamics likely underpin part of these effects. Classic and modern materials research shows a roughness “threshold” near Ra ≈ 0.2 µm: above this, supragingival plaque retention increases steeply, whereas polishing below this threshold yields diminishing microbiologic returns [35]. Surface free energy interacts with roughness—high-energy and rougher substrates bind plaque more strongly and shift composition—but roughness tends to dominate supragingivally [36]. Contemporary in vitro work further confirms that rougher provisional and acrylic materials accumulate more biofilm and can elicit higher cytotoxicity signals than smoother comparators, emphasizing the need for meticulous finishing, polishing, and maintenance of acrylic intaglio surfaces [37].

Taken together, these data support a pragmatic, design-first prevention algorithm. For palatal-coverage devices, pairing standard toothbrushing with plate-specific regimens (validated effervescent or hypochlorite-based cleaners per manufacturer concentrations, or chlorhexidine as indicated) and scheduling “plate-off” ventilation intervals that do not compromise biomechanics should be considered [22,23]. For patients in whom periodontal endpoints are a priority, minimizing palatal coverage or using appliance designs that preserve access and salivary clearance should be considered—an approach consistent with meta-analytic evidence showing superior periodontal indices with less retentive designs [24,28]. In all cases, routine palatal tissue inspection should complement marginal gingival assessment to detect early mucosal changes that—per our model and prior case literature—track closely with palatal biofilm load [27,32,33,34].

### 4.2. Study Limitations

This single-center, cross-sectional study cannot establish causality and may not generalize beyond adolescents or other clinical settings. Consecutive recruitment can introduce selection bias. Aerobic culture underestimates obligate anaerobes and fungi; although trends were consistent with molecular *S. mutans* data, broader sequencing (16S/ITS) would refine taxonomic resolution. Despite examiner calibration (weighted κ = 0.82 for erythema; ICC ≥ 0.89 for PI/GI), semi-quantitative scoring remains susceptible to observer bias. Residual confounding is possible (e.g., behaviors not captured by our brief questionnaire), although models adjusted for wear duration, stage, parental education, and baseline DMFT and results were stable in sensitivity analyses. Participants were recruited consecutively at a single university clinic, which may limit generalizability to other settings or adult populations.

## 5. Conclusions

Within the constraints of a cross-sectional design, appliance type was associated with distinct colonization patterns and soft-tissue responses in adolescents, with palate-contacting acrylic components showing higher palatal biofilm and erythema than bands. These associations support pragmatic, device-specific hygiene counseling and routine palatal inspection as hypothesis-generating strategies pending longitudinal confirmation.

## Figures and Tables

**Table 1:** Baseline characteristics by device group.

Group	Age (y)	Female (%)	Wear Duration (Months)	Toothbrushing (Times/Day)	Sugary Drinks (Times/Week)	Treatment Stage, n (%)	Parental Education >Secondary, %	Baseline DMFT, Mean ± SD
Band (n = 44)	15.3 ± 1.9	45.5	7.4 ± 2.9	2.1 ± 0.4	4.1 ± 1.8	Leveling 21 (47.7); Space-closure 15 (34.1); Finishing 8 (18.2)	56.8	2.1 ± 1.2
Nance (n = 41)	14.6 ± 1.6	63.4	9.6 ± 3.8	2.1 ± 0.4	4.4 ± 1.9	Leveling 15 (36.6); Space-closure 18 (43.9); Finishing 8 (19.5)	51.2	2.3 ± 1.3
Acrylic (n = 43)	14.9 ± 2.1	46.5	5.6 ± 2.6	2.2 ± 0.4	3.9 ± 2.1	Leveling 18 (41.9); Space-closure 15 (34.9); Finishing 10 (23.2)	58.1	2.0 ± 1.1
Overall *p*-value	0.261	0.182	<0.001	0.371	0.410	0.642	0.712	0.568

*p*-values from ANOVA or χ^2^ as appropriate. DMFT, decayed-missing-filled teeth; “Parental education” used as SES proxy.

**Table 2:** Microbial load by device and site.

Group	Device Biofilm (log10 CFU/cm^2^)	Adjacent Enamel (log10 CFU/cm^2^)	Palatal Mucosa (log10 CFU/cm^2^)	*S. mutans* Load (log10 copies/mL)
Band	4.6 ± 0.6	3.7 ± 0.6	3.2 ± 0.4	5.2 ± 0.6
Nance	5.3 ± 0.6	4.3 ± 0.4	4.8 ± 0.6	5.9 ± 0.7
Acrylic	5.6 ± 0.6	4.6 ± 0.6	4.9 ± 0.7	6.2 ± 0.7

Overall *p* (ANOVA): device biofilm < 0.001; adjacent enamel < 0.001; palatal mucosa < 0.001; *S. mutans* < 0.001.

**Table 3:** Periodontal and palatal soft-tissue outcomes.

Group	Plaque Index (0–3)	Gingival Index (0–3)	Bleeding on Probing (%)	Probing Depth (mm)	Palatal Erythema (0–3)
Band	1.6 ± 0.3	1.2 ± 0.3	22.3 ± 11.3	2.6 ± 0.4	0.6 ± 0.4
Nance	2.1 ± 0.2	1.7 ± 0.3	32.9 ± 13.9	2.9 ± 0.4	1.6 ± 0.8
Acrylic	1.7 ± 0.3	1.6 ± 0.3	28.9 ± 12.2	2.8 ± 0.6	1.9 ± 0.7

Overall *p* (ANOVA): PI < 0.001; GI < 0.001; BOP% 0.001; PD 0.015; palatal erythema < 0.001.

**Table 4:** Subgroup comparison: fixed vs. removable (pooled analysis).

Group Type	Palatal Mucosa (log10 CFU/cm^2^)	Palatal Erythema (0–3)	Gingival Index (0–3)
Fixed (bands + Nance)	4.1 ± 0.9	1.1 ± 0.8	1.6 ± 0.4
Removable (acrylic)	4.9 ± 0.7	1.9 ± 0.7	1.6 ± 0.3

Between-group *p* (Welch t-test): palatal CFU < 0.001; palatal erythema < 0.001; GI 0.082.

**Table 5:** Correlations between colonization and clinical outcomes (Spearman).

Correlation	Spearman ρ	*p*-Value
CFU_Device vs. GI	0.3	<0.001
CFU_Device vs. BOP%	0.1	0.359
CFU_Device vs. PD_mm	0.2	0.029
CFU_Device vs. Palatal Erythema	0.4	<0.001
CFU_Device vs. Wear months	0.1	0.993
CFU_Device vs. SSB/week	0.1	0.106
CFU_Device vs. Brush/day	0.1	0.838
Palatal CFU vs. Erythema	0.6	<0.001
*S. mutans* vs. *GI*	0.3	0.003

**Table 6:** Multivariable model for gingival inflammation (dependent variable: Gingival Index).

Term	Beta	95% CI	*p*-Value
Intercept	0.98	0.22 to 1.74	0.013
Device: Acrylic vs. Band	+0.38	+0.20 to +0.56	<0.001
Device: Nance vs. Band	+0.52	+0.33 to +0.71	<0.001
Device biofilm (per log_10_ CFU)	+0.06	−0.06 to +0.18	0.327
Toothbrushing (per time/day)	−0.03	−0.16 to +0.10	0.637
Sugary drinks (per time/week)	+0.02	−0.02 to +0.06	0.335
Age (per year)	+0.01	−0.03 to +0.05	0.585
Female (vs. male)	+0.04	−0.07 to +0.15	0.493
Wear duration (per month)	+0.02	−0.01 to +0.04	0.164
Treatment stage: Space-closure vs. Leveling	+0.08	−0.06 to +0.22	0.258
Treatment stage: Finishing vs. Leveling	+0.05	−0.12 to +0.22	0.566
Parental education (>secondary)	−0.06	−0.17 to +0.05	0.278
Baseline DMFT (per unit)	+0.04	−0.02 to +0.10	0.179

HC3-robust OLS; R^2^ = 0.33. Covariates were prespecified. Device coefficients remain significant after additional adjustment (stage, SES, DMFT).

## Data Availability

Data available on request.

## References

[1] Luchian I., Surlari Z., Goriuc A., Ioanid N., Zetu I., Butnaru O., Scutariu M.M., Tatarciuc M., Budala D.G. (2024). The Influence of Orthodontic Treatment on Periodontal Health between Challenge and Synergy: A Narrative Review. Dent. J..

[2] Widhianingsih D., Koontongkaew S. (2021). Enhancement of cariogenic virulence properties of dental plaque in asthmatics. J. Asthma.

[3] Niu Q., Chen S., Bai R., Lu Y., Peng L., Han B., Yu T. (2024). Dynamics of the oral microbiome during orthodontic treatment and antimicrobial advances for orthodontic appliances. iScience.

[4] Maret D., Marchal-Sixou C., Vergnes J.N., Hamel O., Georgelin-Gurgel M., Van Der Sluis L., Sixou M. (2014). Effect of fixed orthodontic appliances on salivary microbial parameters at 6 months: A controlled observational study. J. Appl. Oral Sci..

[5] Thanetchaloempong W., Koontongkaew S., Utispan K. (2022). Fixed Orthodontic Treatment Increases Cariogenicity and Virulence Gene Expression in Dental Biofilm. J. Clin. Med..

[6] Shokeen B., Viloria E., Duong E., Rizvi M., Murillo G., Mullen J., Shi B., Dinis M., Li H., Tran N.C. (2022). The impact of fixed orthodontic appliances and clear aligners on the oral microbiome and the association with clinical parameters: A longitudinal comparative study. Am. J. Orthod. Dentofac. Orthop..

[7] Gong W., Zhou K., Li S., Yue Z., Zhang Q., Li Y., Mi X. (2025). Different Effects of Fixed Appliances and Clear Aligners on the Microbiome and Metabolome of Dental Plaque. Orthod. Craniofacial Res..

[8] Yáñez-Vico R.M., Iglesias-Linares A., Ballesta-Mudarra S., Ortiz-Ariza E., Solano-Reina E., Perea E.J. (2015). Short-term effect of removal of fixed orthodontic appliances on gingival health and subgingival microbiota: A prospective cohort study. Acta Odontol. Scand..

[9] Catunda R.Q., Altabtbaei K., Flores-Mir C., Febbraio M. (2023). Pre-treatment oral microbiome analysis and salivary Stephan curve kinetics in white spot lesion development in orthodontic patients wearing fixed appliances. A pilot study. BMC Oral Health.

[10] Kado I., Hisatsune J., Tsuruda K., Tanimoto K., Sugai M. (2020). The impact of fixed orthodontic appliances on oral microbiome dynamics in Japanese patients. Sci. Rep..

[11] Alrehaili R., Alhujaili A., Almanjhi W., Alnami H., Alsaiyari S., Alqahtani H., Alabdan R., Baamer D., Khalil A. (2024). How Effective Are the Nance Appliance and Transpalatal Arch at Reinforcing Anchorage in Extraction Cases?. Cureus.

[12] Patini R., Bonetti A.A., Camodeca A., Staderini E., Gallenzi P. (2018). Haematemesis related to orthodontic treatment with Nance palatal arch: A case report. J. Orthod..

[13] Pellissari B.A., Sabino G.S.P., de Souza Lima R.N., Motta R.H.L., Suzuki S.S., Garcez A.S., Basting R.T., Barbosa J.A., Martins Montalli V.A. (2021). Antimicrobial resistance of bacterial strains in patients undergoing orthodontic treatment with and without fixed appliances. Angle Orthod..

[14] Jing D., Hao J., Shen Y., Tang G., Lei L., Zhao Z. (2019). Effect of fixed orthodontic treatment on oral microbiota and salivary proteins. Exp. Ther. Med..

[15] Arab S., Nouhzadeh Malekshah S., Abouei Mehrizi E., Ebrahimi Khanghah A., Naseh R., Imani M.M. (2016). Effect of Fixed Orthodontic Treatment on Salivary Flow, pH and Microbial Count. J. Dent..

[16] Löe H. (1967). The Gingival Index, the Plaque Index and the Retention Index Systems. J. Periodontol..

[17] Ainamo J., Bay I. (1975). Problems and proposals for recording gingivitis and plaque. Int. Dent. J..

[18] Yano A., Kaneko N., Ida H., Yamaguchi T., Hanada N. (2002). Real-time PCR for quantification of Streptococcus mutans. FEMS Microbiol. Lett..

[19] Bustin S.A., Benes V., Garson J.A., Hellemans J., Huggett J., Kubista M., Mueller R., Nolan T., Pfaffl M.W., Shipley G.L. (2009). The MIQE guidelines: Minimum information for publication of quantitative real-time PCR experiments. Clin. Chem..

[20] Von Elm E., Altman D.G., Egger M., Pocock S.J., Gøtzsche P.C., Vandenbroucke J.P., STROBE Initiative (2007). The Strengthening the Reporting of Observational Studies in Epidemiology (STROBE) statement: Guidelines for reporting observational studies. Ann. Intern. Med..

[21] Pathak A.K., Sharma D.S. (2013). Biofilm associated microorganisms on removable oral orthodontic appliances in children in the mixed dentition. J. Clin. Pediatr. Dent..

[22] Charavet C., Graveline L., Gourdain Z., Lupi L. (2021). What Are the Cleaning and Disinfection Methods for Acrylic Orthodontic Removable Appliance? A Systematic Review. Children.

[23] Khawwam S.I., Al-Groosh D.H. (2023). Effect of Different Cleaning Regimes on Biofilm Formation of Acrylic-Based Removable Orthodontic Appliance: A Randomized Clinical Trial. Sci. World J..

[24] Crego-Ruiz M., Jorba-García A. (2023). Assessment of the periodontal health status and gingival recession during orthodontic treatment with clear aligners and fixed appliances: A systematic review and meta-analysis. Med. Oral Patol. Oral Cir. Bucal.

[25] Jiang Q., Li J., Mei L., Du J., Levrini L., Abbate G.M., Li H. (2018). Periodontal health during orthodontic treatment with clear aligners and fixed appliances: A meta-analysis. J. Am. Dent. Assoc..

[26] Yu G., Wang J., Lin X., Diao S., Cao Y., Dong R., Wang L., Wang S., Fan Z. (2016). Demethylation of SFRP2 by histone demethylase KDM2A regulated osteo-/dentinogenic differentiation of stem cells of the apical papilla. Cell Prolif..

[27] Mummolo S., Nota A., Albani F., Marchetti E., Gatto R., Marzo G., Quinzi V., Tecco S. (2020). Salivary levels of Streptococcus mutans and Lactobacilli and other salivary indices in patients wearing clear aligners versus fixed orthodontic appliances: An observational study. PLoS ONE.

[28] Sifakakis I., Papaioannou W., Papadimitriou A., Kloukos D., Papageorgiou S.N., Eliades T. (2018). Salivary levels of cariogenic bacterial species during orthodontic treatment with thermoplastic aligners or fixed appliances: A prospective cohort study. Prog. Orthod..

[29] Paterlini M. (2024). Italy toughens sanctions against perpetrators of violent attacks on hospital staff. BMJ.

[30] Cakmak F., Turk T., Karadeniz E.I., Elekdag-Turk S., Darendeliler M.A. (2014). Physical properties of root cementum: Part 24. Root resorption of the first premolars after 4 weeks of occlusal trauma. Am. J. Orthod. Dentofac. Orthop..

[31] Marda A., Elhamzaoui S., El Mansari A., Souly K., Farissi F., Zouhdi M., Zaoui F., Bahije L. (2018). Evaluation of Changes in Cariogenic Bacteria in a Young Moroccan Population with Fixed Orthodontic Appliances. Int. J. Dent..

[32] Zhao S., Rogers M.J., He J. (2017). Microbial reductive dehalogenation of trihalomethanes by a Dehalobacter-containing co-culture. Appl. Microbiol. Biotechnol..

[33] Seethamraju H., Paul S. (2018). Wrap it up! Should we take it?. J. Thorac. Cardiovasc. Surg..

[34] Sheikh H.K., Arshad T., Merajoddin M., Mohammad Z.S., Usman R., Hasan M.M. (2020). Docking analysis of aryl derivatives of diepoxide alkylating agents. Pak. J. Pharm. Sci..

[35] Bollen C.M., Lambrechts P., Quirynen M. (1997). Comparison of surface roughness of oral hard materials to the threshold surface roughness for bacterial plaque retention: A review of the literature. Dent. Mater..

[36] Quirynen M., Bollen C.M. (1995). The influence of surface roughness and surface-free energy on supra- and subgingival plaque formation in man. A review of the literature. J. Clin. Periodontol..

[37] Plante-Hébert J., Boucher V.J., Jemel B. (2021). The processing of intimately familiar and unfamiliar voices: Specific neural responses of speaker recognition and identification. PLoS ONE.

